# Clinical experience with adaptive MRI-guided pancreatic SBRT and the use of abdominal compression to reduce treatment volume

**DOI:** 10.3389/fonc.2024.1441227

**Published:** 2024-08-09

**Authors:** William S. Ferris, Benjamin George, Kristin A. Plichta, Joseph M. Caster, Daniel E. Hyer, Blake R. Smith, Joel J. St-Aubin

**Affiliations:** Department of Radiation Oncology, University of Iowa, Iowa, IA, United States

**Keywords:** MR-linac, Unity, compression, adaptive, SBRT, pancreatic cancer

## Abstract

**Introduction:**

This work presents a method to treat stereotactic body radiation therapy (SBRT) for pancreatic cancer on a magnetic resonance-guided linear accelerator (MR-linac) using daily adaptation, real-time motion monitoring, and abdominal compression.

**Methods:**

The motion management and treatment planning process involves a magnetic resonance imaging (MRI) simulation with cine and 3D images, a computed tomography (CT) simulation with a breath-hold CT and a 4DCT, pre-treatment verification and planning MRI, and intrafraction MRI cine images.

**Results:**

The results from 26 patients were included in this work. Our motion management process results in consistent motion analysis on the CT simulation, MRI simulation, and each treatment fraction. The liver dome was found to be an overestimate of tumor superior/inferior (SI) motion for most patients. Adding compression reduced SI liver dome motion by 6.2 mm on average. Clinical outcomes are similar to those observed in the literature.

**Conclusions:**

In this work, we demonstrate how pancreatic SBRT can be successfully treated on an MR-linac using abdominal compression. This allows for an increased duty cycle compared to gating and/or breath-hold techniques.

## Introduction

Pancreatic cancer (and metastases located in the pancreas) is often unresectable, and there is growing interest in utilizing dose-escalated stereotactic body radiation therapy (SBRT) to provide better overall local control over these lesions. Dose-escalated SBRT is enabled with advanced motion management and imaging techniques to reduce margins and accurately locate the target. Pancreatic treatments are also complicated by respiratory motion due to the location of the pancreas in the abdomen. For example, the pancreas is observed to move an average of 20 mm for shallow breaths and 43 mm for deep breaths (1). This motion must be managed if small margins are desired. In addition, isotoxic treatment planning is often used for pancreatic SBRT because the location of organs at risk (OARs) limits the target coverage that can be achieved for each fraction (2).

Magnetic resonance (MR)-guided linear accelerators (linacs) provide enhanced soft tissue contrast on pre-treatment images, which allows for daily adaptation of the treatment plan based on anatomical variations (3, 4). In addition, MR-linacs provide the ability to visualize the target during the treatment without additional ionizing radiation dose. This allows for real-time gating of the treatment, such as on the ViewRay MRIdian or more recently the Elekta Unity (3, 5). However, gating can result in a reduced duty cycle since the beam is only on when the tumor is in a desired phase of respiration. For tight margins with large respiratory motion, the duty cycle may be prohibitively small, leading to long treatment times on top of an already long treatment process with adaptive planning. Breath holds are commonly used in pancreatic treatments, but these may be uncomfortable or impossible for some patients to perform. Breath-hold techniques also inherently have a lower duty cycle due to the need for the patient to recover between breath holds.

Patients accrued to this study were treated prior to Elekta’s release of the comprehensive motion management (CMM) system on the Unity, which allows for real-time tumor tracking and gating including breath hold. The results presented in this work focus on the use of abdominal compression, which would still be a viable option when coupled with gating in CMM to physically reduce total motion. The application of gating with compression would serve to further reduce the motion vector during treatment, which would in turn improve the duty cycle.

Several studies have presented clinical results for MR-guided pancreatic treatments and attempted to standardize planning techniques for these treatments (6–9). However, these studies have been multi-institutional and have not had uniform motion management strategies, imaging techniques, and workflows. This work presents specific clinical workflows that can be reproduced by other clinics attempting to begin these treatments.

The purpose of this work is to share our experience with SBRT to the pancreas on the Unity MR-linac (Elekta, Stockholm, Sweden) without the use of breath hold and gating (4). Small margins are achieved through the use of daily adaptation, real-time cine motion monitoring, and abdominal compression to reduce motion for some of our patients.

## Materials and methods

This human subject research was reviewed and approved by the University of Iowa IRB-01 (Biomedical, application 201701826). The informed consent requirement was waived as allowed under 45 CFR 46. The research was conducted in compliance with ICH E6(R2) as adopted by U.S. law.

The workflow used by our institution for the treatment of pancreatic SBRT on the Unity, including simulation, motion management, and treatment delivery, is described in detail. The results of the workflow were analyzed using 26 patients with pancreatic adenocarcinoma treated in our clinic. All patients were treated with five-fraction SBRT on the Unity with a prescribed dose between 32.5 Gy and 50 Gy. Commissioning and quality assurance processes used in our clinic for the Unity have been described in the literature (10–12).

The imaging procedures used in the workflow are summarized in **Table 1**. Simulation images are acquired with both a Siemens Biograph (Munich, Germany) computed tomography (CT) scanner and a Siemens Vida 3T magnetic resonance imaging (MRI) scanner. Patients treated on the Unity MR-linac are simulated using MRI, which includes MRI cine images, T1-weighted images, and T2-weighted images. The first step in the MRI simulation process is to determine the range of motion of the target, which is used to determine whether abdominal compression is necessary to reduce tumor motion. Sagittal/coronal cine MRI images centered on the dome of the liver are acquired and used to measure the motion of the liver dome in the superior/inferior (SI) direction. The SI motion of the liver dome is used as a surrogate (typically an overestimate) of the target SI motion. If the liver dome SI motion is 15 mm or larger, abdominal compression is added. Compression is added using a Freedom X belt (CDR Systems, Calgary, AB, Canada), and liver dome motion is re-analyzed post-compression with MRI cine. The compression belt size and pressure are stored and repeated at the time of 4DCT simulation and each treatment fraction.

**Table 1:** Image guidance procedures used for pancreatic SBRT treatments on the Unity.

Step	Description	Device	Purpose
1	MRI cine	Siemens Vida 3T	Liver dome motion evaluation
2*	MRI cine	Siemens Vida 3T	Liver dome motion evaluation (post-compression)
3	MRI T1 and T2	Siemens Vida 3T	Secondary planning/contouring images
4	MRI cine	Siemens Vida 3T	Tumor motion evaluation
5	CT breath hold	Siemens Biograph CT	Primary planning CT
6	4DCT	Siemens Biograph CT	Tumor motion evaluation
7	MRI 3D	Unity 1.5T	Adaptive planning before each fraction
8*	MRI cine	Unity 1.5T	Liver dome motion evaluation before each fraction
9	MRI cine	Unity 1.5T	Passive intrafraction tumor position monitoring

SBRT, stereotactic body radiation therapy; CT, computed tomography.

*Only performed if compression is applied.

The next step in simulation is to acquire volumetric T1- and T2-weighted images. The T2-weighted image is acquired first, which is a navigator-triggered sequence acquired at exhale and without contrast. The navigator-triggered sequences use a 1D navigator scan through the liver to determine respiratory motion. The 3D image is captured in segments when the navigator sequence determines that the patient is in their expiration breathing phase. Next, a series of T1-weighted images are acquired at various time points relative to the injection of Gadavist (Bayer, Leverkusen Germany), a gadolinium-based contrast, specifically 1) pre-contrast, 2) arterial phase, 3) venous phase, and 4) a delayed phase waiting 5 minutes after injection. These images assist in the delineation of the tumor and OARs. The last step in the MRI simulation is repeating the acquisition of sagittal/coronal cine images but centered over the tumor instead of the liver dome. This is performed after the volumetric images to provide better localization of the target planes on the tumor. These images are used to check whether the liver dome is a good surrogate of motion for the tumor.

The last step in simulation is the acquisition of exhale breath hold CT and 4DCT images. The internal target volume (ITV) is defined as the summation of the gross tumor volume (GTV) on the exhale breath hold CT and the GTV at end-expiration and inhalation of the 4DCT scan (ITV = GTV_BH + GTV_0EX + GTV_100IN). The planning target volume (PTV) is typically a 5-mm expansion of the ITV.

All treatment planning is performed using the Monaco treatment planning system (TPS; Elekta, Stockholm, Sweden) (2). In terms of our planning technique, our initial institutional practice was to select a dose that respected normal bowel constraints while maintaining target coverage. Our practice has since evolved to prescribe to a high dose level (50 Gy in five fractions) using an isotoxic approach where target coverage was determined by dose to adjacent structures, similar to that of the stereotactic MR-guided adaptive radiotherapy (SMART) trial (6). Planning organ at risk volumes (PRVs) are used when applicable. For most patients, the dose-limiting OAR constraints are stomach/duodenum/small bowel V33Gy < 0.5 cc. Other constraints used at our institution are large bowel V35Gy < 0.5 cc, liver V12Gy < 50%, kidneys V12Gy < 75%, spinal cord D1cc < 20 Gy, and great vessel D0.03cc < 53 Gy. The gut, defined as the combination of the stomach, duodenum, large bowel, and small bowel structures, was the focus of this work since it was the dose-limiting structure for most cases.

Pre-treatment MRI images used for adaptive planning on the Unity are either balanced contrast 3D-VANE or T2 nav-triggered sequences. A sagittal/coronal cine is acquired centered over the liver dome prior to each fraction to ensure that the motion (and applied compression) is consistent with the simulation. In addition, a sagittal/coronal cine is also acquired during each fraction centered on the target to monitor the target position passively throughout the entire fraction. If the target is observed to move outside the PTV, the treatment is manually paused.

There are two types of adaptive techniques on the Unity: adapt to position (ATP) and adapt to shape (ATS). Each plan must be adapted since the Unity only has SI couch motion. ATP is analogous to couch shifts on a conventional linac (13). The pre-treatment image is aligned with the planning image, the multileaf collimator (MLC) pattern is shifted based on the updated target position in the bore, and the beams are re-optimized to match the original target dose coverage and other dose volume histogram metrics for the organs at risk. For ATS, the contours are modified, and a fully re-optimized plan is created based on the new contours. To evaluate the effect of isotoxic planning, the overlap volume between the gut and the PTV is tabulated for the reference plan and each fraction for all patients.

We retrospectively identified 30 patients who were treated with SBRT to the pancreas at the time of radiation using this protocol between October 2019 and February 2023. Two patients were excluded due to not completing their entire treatment course during the institutional review board (IRB)-determined period. One patient was excluded due to not finishing their treatment course. Another patient was excluded because one of five fractions was delivered using a non-MR-linac due to equipment downtime. A total of 26 patients were included in this study with IRB approval.

## Results

A summary of the patient treatment statistics is shown in **Table 2**. Twenty-three subjects had pancreatic ductal adenocarcinoma (PDAC), and three were treated for pancreatic metastases (one colorectal cancer patient and two renal cell carcinoma). Among PDAC patients, 88% had primary pancreatic ductal adenocarcinoma tumors, and 12% were treated for vascular recurrences after prior Whipple resection. Most PDAC patients (76%) received chemotherapy before and/or after SBRT, although 24% received palliative SBRT without any chemotherapy. Three subjects were on a trial that randomized them to receive the SOD mimic GC47111 concurrent with SBRT (NCT04698915). The prescription varied from 32.5 Gy to 50 Gy, with an average of 41.4 Gy. The average PTV coverage at the prescription isodose line was 77.9% [min 31.0%, max 95.9%]. The average “PTV minus gut PRV” coverage at the 32 Gy isodose line was 96.3% [83.5%, 100.0%].

**Table 2:** Summary of patient statistics.

Patient	Prescribed dose (Gy)	Compression	# fx ATP/ATS	PTV vol (cc)	PTV V_Rx_ (%)	PTV-GutPRV V_32.5Gy_ (%)
1	36.0	No	2/3	63.2	79.8	96.6
2	37.5	No	0/5	30.6	95.7	100.0
3	35.0	No	3/2	32.4	95.9	99.9
4	35.0	No	1/4	106.5	92.3	100.0
5	45.0	No	5/0	84.0	95.1	100.0
6	40.0	No	4/1	7.4	95.0	100.0
7	45.0	No	4/1	44.4	86.8	99.5
8	40.0	No	4/1	27.4	95.9	100.0
9	35.0	No	5/0	23.1	79.5	95.7
10	32.5	No	4/1	64.3	79.7	84.8
11	32.5	No	5/0	48.5	94.6	97.2
12	35.0	No	1/4	45.7	77.7	97.1
13	50.0	No	0/5	61.3	67.6	97.7
14	50.0	Yes	0/5	138.5	52.9	96.1
15	40.0	Yes	0/5	120.4	31.0	83.5
16	50.0	No	0/5	144.8	92.1	86.7
17	45.0	Yes	0/5	81.6	45.2	92.2
18	50.0	Yes	0/5	104.2	84.7	99.9
19	40.0	Yes	0/5	32.0	53.4	97.6
20	40.0	Yes	0/5	35.5	95.1	100.0
21	35.0	No	0/5	145.5	84.0	95.3
22	45.0	Yes	0/5	59.6	84.4	99.4
23	50.0	Yes	0/5	127.6	61.0	97.0
24	50.0	Yes	0/5	125.8	55.6	95.8
25	50.0	Yes	0/5	117.5	60.9	98.5
26	33.0	Yes	0/5	62.8	88.5	96.6
Avg	41.4	n/a	n/a	74.7	77.9	96.3

All prescriptions are for five fractions. PTV volumes and coverage are for the reference plan only.

PTV, planning target volume.

**Figure 1:**
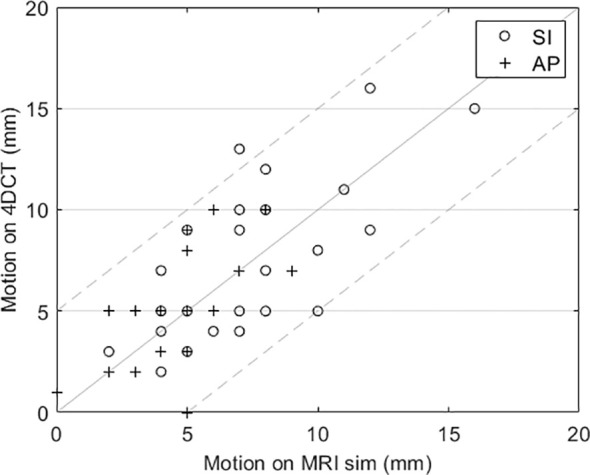
Comparison of tumor motion evaluated using cine MRI and 4DCT for each patient. The dashed lines indicate the ±5 mm desired agreement between MRI and 4DCT motion.

The motion management results were used to validate various aspects of our treatment process. First, a comparison of tumor motion between the MRI sim and CT sim is shown in **Figure 1**. For all but one patient, the tumor motion on the CT (see step 6 in **Table 1**) was within ±5 mm of the motion evaluated on MRI (step 4). If the tumor motion has a difference of more than 5 mm between the modalities, this is an indication of inconsistency such as respiratory pattern changes or belt setting changes and is investigated.

**Figure 2:**
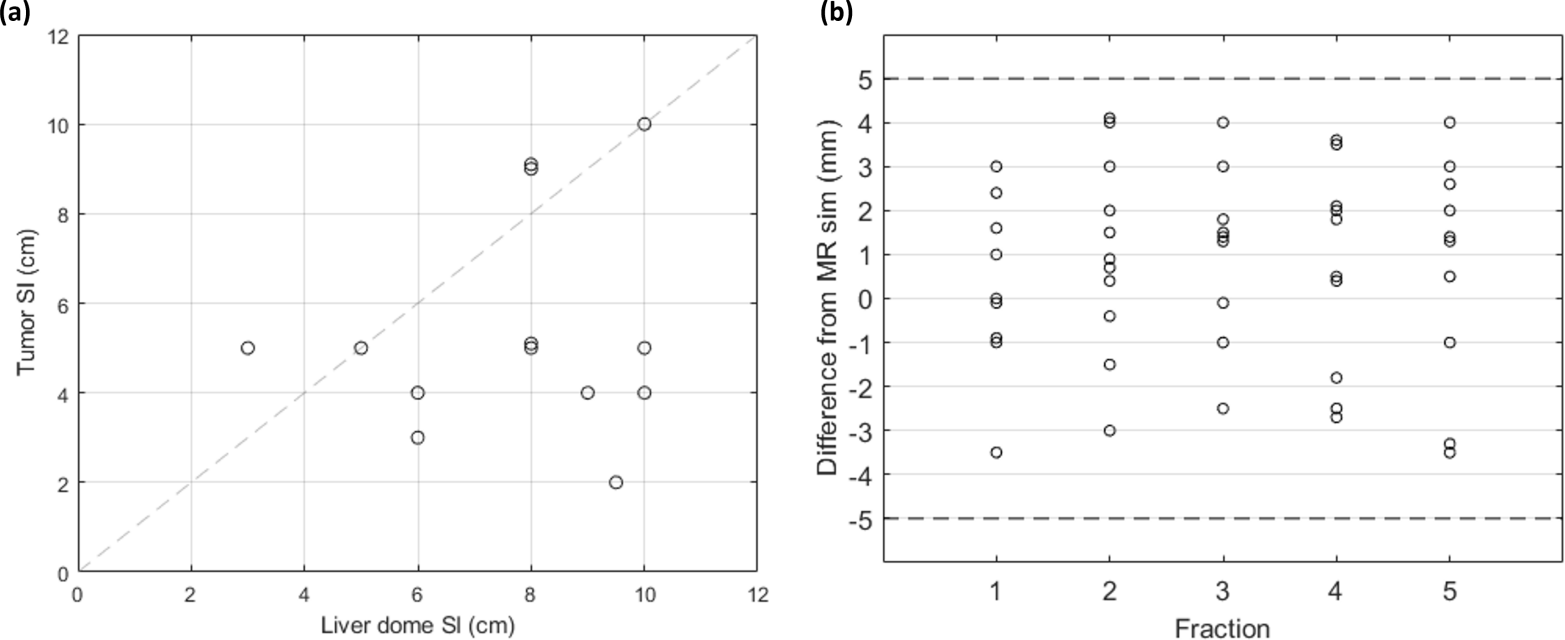
**(A)** SI motion of the liver dome and tumor for patients with compression (n = 11), analyzed using MRI cine. **(B)** Difference in SI liver dome motion between each treatment fraction and at MRI sim. The dashed lines indicate the ±5 mm desired agreement between sim and each fraction. SI, superior/inferior; MRI, magnetic resonance imaging.

The next motion validation step was to determine how well the SI motion of the liver dome represents the motion of the tumor. **Figure 2A** displays the SI motion of the liver dome versus the motion of the tumor. The liver dome was an overestimate of tumor motion for all but three cases. The average difference in motion between the tumor and liver is −2.6 mm [−7.5 mm, 2 mm], indicating that tumor motion is typically less than liver dome motion. **Figure 2B** indicates how the liver dome motion evaluated on each fraction was within ±5 mm of the motion observed during the simulation for all fractions.

**Figure 3:**
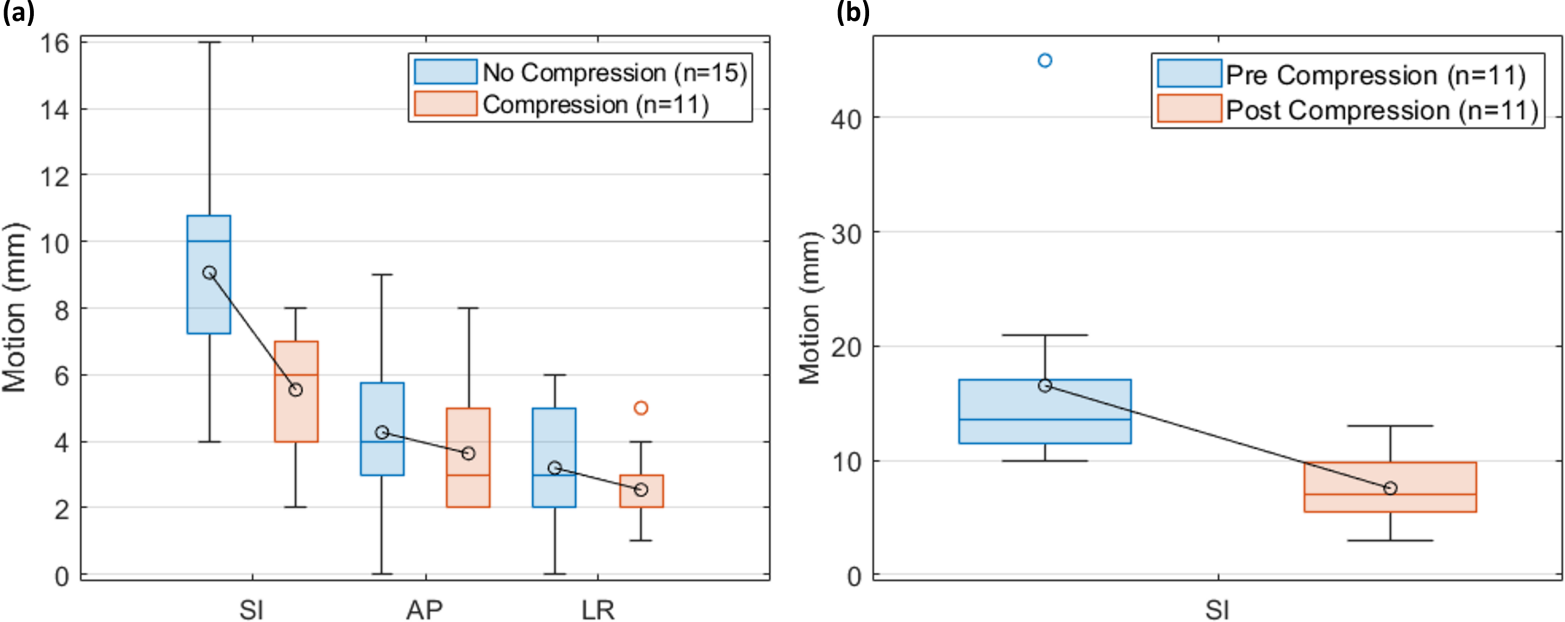
The effect of compression. **(A)** Comparison of tumor motion using 4DCT for the patients with (n = 11) and without (n = 15) compression. **(B)** Comparison of liver dome motion using MRI for the compressed patients (n = 11). The boxplot indicates the first, second, and third quartiles. Error bars indicate the non-outlier minima and maxima. Averages are indicated in the circles.

The effect of adding abdominal compression is shown in **Figure 3**. **Figure 3A** compares the tumor motion between the compressed and non-compressed patient cohorts. On average, the non-compressed patient tumor motion magnitudes were 9.1 mm, 4.3 mm, and 3.2 mm in the SI, anterior-posterior (AP), and left-right (LR) directions, respectively, and the compressed patient tumor motion magnitudes were 5.5 mm, 3.6 mm, and 2.5 mm, respectively. A two-sided t-test indicates a significant difference in the 3D motion vector (p = 0.002). **Figure 3B** compares the pre- and post-compression liver dome SI motion for the compressed patients. On average, the liver dome SI motion decreased from 13.9 mm [10 mm, 45 mm] without compression to 7.7 mm [3 mm, 13 mm] with compression, a significant decrease (p = 0.008).

**Figure 4:**
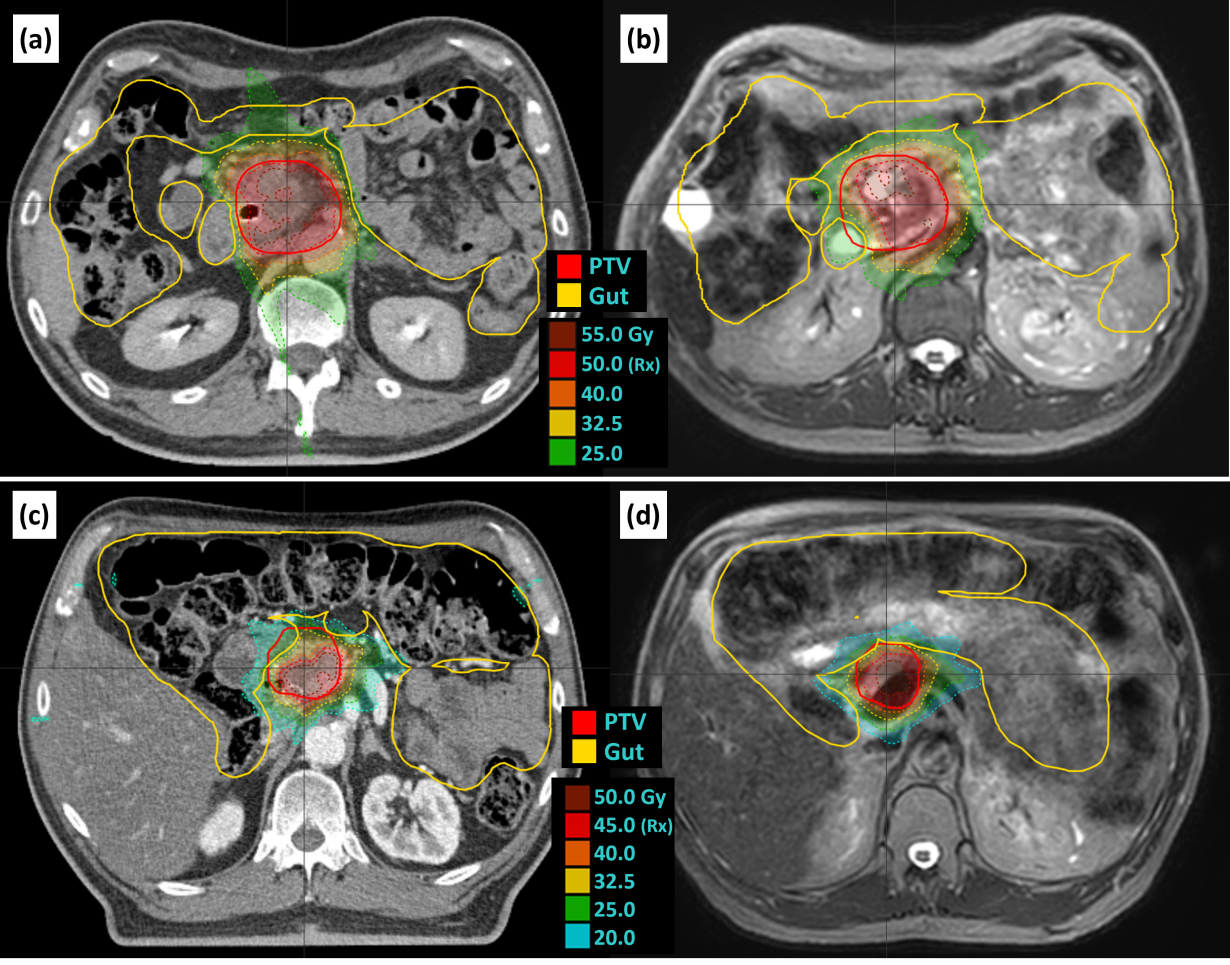
Examples of isotoxic planning. Patient 8 reference plan **(A)** and fraction 1 **(B)** as an example of increased overlap and decreased coverage relative to the reference plan. Patient 17 reference plan **(C)** and fraction 3 **(D)** as an example of decreased overlap and increased coverage. Panels A and C are reference plans on the reference CT, and panels B and D are example adapted plans on the daily T2 navigator-triggered MRI. CT, computed tomography; MRI, magnetic resonance imaging.

Two examples of isotoxic planning are shown in **Figure 4**. For patient 8, the overlap volume of the PTV and the gut was 0.2 cc for the reference plan and 7.9 cc for fraction 1, leading to a decrease in PTV coverage from 84.7% to 52.7%. For patient 17, the overlap of the PTV and the gut was 3.3 cc for the reference plan and 0.1 cc for fraction 1, which allowed for an increase in coverage from 45.2% to 67.4%.

**Figure 5:**
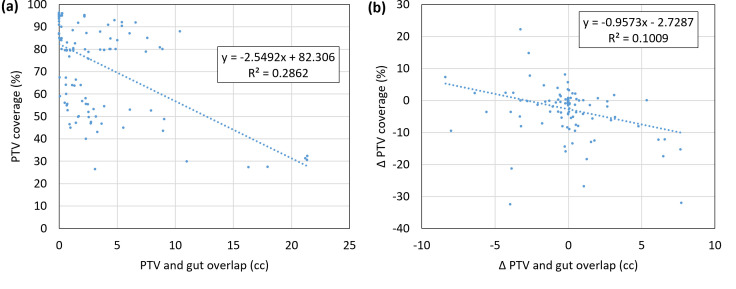
**(A)** Correlation of PTV coverage and the overlap volume of the PTV and gut. **(B)** Change in coverage and change in volume relative to the reference plan. Data points are shown for all adapted fractions for all patients (n = 5 * 26 = 130). PTV, planning target volume.

The effect of isotoxic planning can also be demonstrated by analyzing the change in coverage corresponding to changes in overlap volumes with OARs. **Figure 5A** shows the relationship between the overlap volume of the gut and PTV, versus PTV coverage while **Figure 5B** shows the change in coverage and change in volume relative to the reference plan. Although the linear correlation is not significant, a relationship can be observed that as the overlap volume becomes large, the coverage decreases. For isotoxic planning, it is expected that if the overlap volume increases relative to the reference plan, coverage will decrease. When the overlap volume was zero, the PTV coverage was 85% or better.

**Table 3** shows clinical outcomes. The mean follow-up from the completion of radiation therapy was 8.3 months. At the time of the last follow-up, eight patients were alive with no progression, two patients were alive with distant progression, and 16 patients had died. SBRT resulted in a good rate of local control, with five of 26 patients (19%) having local progression, with a median of 5.6 months to local progression. Fourteen patients had metastatic progression, with a median of 3.1 months to metastatic progression. SBRT was generally well-tolerated. There were three patients with grade 3 or higher toxicities that may be related to SBRT. Two patients developed Gastrointestinal (GI) hemorrhage that may be related to prior SBRT treatment.

**Table 3:** Clinical outcomes from SBRT.

Best local response to SBRT								
CR				1 (3.8%)				
SD				12 (46.2%)				
PR				7 (26.9%)				
PD				5 (19.2%)				
Local progression								
Yes				5 (19.2%)				
No				21 (80.8%)				
Time to local progression (med)				5.6 months (1.5–11.9)				
Metastatic progression								
Yes				14 (53.8%)				
No				12 (46.2%)				
Time to metastatic progression (med)				3.1 months (0.8–17.7)				
Maximum grade toxicity: N (%)								
CTCAE item	0	1		2	3	4	5	
Nausea	15 (54.1)	2 (7.7)		9 (34.6)	-	-	-	
Abdominal pain	12 (46.1)	7 (26.9)		6 (23.1)	-	-	-	
Anorexia	19 (73.1)	5 (19.2)		2 (7.7)	-	-	-	
Diarrhea	21 (80.8)	3 (11.5)		2 (7.7)	-	-	-	
Constipation	24 (92.3)	-		2 (7.7)	-	-	-	
Gastric/duodenal ulceration*	24 (92.3)	-		2 (7.7)	-	-	-	
GI hemorrhage	24 (92.3)	-		-	1 (3.8)	-	1 (3.8)	
liver function test (LFT) increased	16 (61.5)	8 (30.7)		2 (7.7)	-	-	-	
Infection (cholangitis)	23 (88.4)	-		-	3 (11.5)	-	-	
Fatigue	13 (50)	11 (42.3)		2 (7.7)	-	-	-	
Back pain	22 (84.5)	4 (15.3)		-	-	-	-	
Dizziness	23 (88.4)	2 (7.7)		1 (3.8)	-	-	-	

CR, complete response; SD, stable disease; PR, partial response; PD, progressive disease; CTCAE, Common Terminology Criteria for Adverse Events; SBRT, stereotactic body radiation therapy.

*Only assessed in subjects with post-SBRT esophagogastroduodenoscopy (EGD) or endoscopic retrograde cholangiopancreatography (ERCP).

## Discussion

As our clinical team developed more experience, compression was used more often, ATS was used more often as opposed to ATP, and the use of dose escalation increased. For example, when compression is used, the prescription was on average 44.8 Gy, compared to 38.9 Gy when compression was not used. Compression reduces the motion, therefore reducing the size of the ITV and PTV and overlapping volumes with the dose-limiting structures, allowing for the higher prescription dose to be delivered. The total number of fractions delivered with ATP and ATS was 38 and 92, respectively. ATS is more ideal for pancreatic SBRT since it allows for daily recontouring and re-optimizing. For the 14 most recent patients included in this study, all fractions were delivered ATS. ATS is now our standard of practice for pancreas SBRT on Unity.

One downside to using compression over breath holds and/or gating is a larger treated volume, as the entire motion range is treated with compression. For example, adding compression reduced SI motion from an average of 13.9 mm to 7.7 mm. Adding gating could decrease this margin further depending on the choice of the gating envelope (with a reduction in duty cycle), with the ultimate reduction achieved with a breath hold technique. Another downside is decreased comfort for the patient with compression applied. However, compression allows the patient to breathe freely. Lastly, compression is prone to inconsistencies in belt settings and location, which could lead to varying fractional tumor motion. This work proposes a method to verify the proper compression and motion of each fraction and alleviate some of this uncertainty.

Using abdominal compression instead of breath holds increases the duty cycle of treatments, but with an increase of overall treatment volume due to the inclusion of the motion-derived ITV. A typical pancreas SBRT treatment on the Unity has 10 minutes of beam-on time. Therefore, it would take 30 breath holds of 20 seconds for a patient to complete one fraction, which may be challenging for some patients. Although some patients included in this study did not require compression, compression increased patient inclusion for treatment on the Unity by decreasing the motion magnitude to 15 mm or less. For example, five of the 11 patients treated with compression had liver dome motion greater than 15 mm, and as large as 45 mm, before compression.

Similar target coverage was achieved in this study as observed in the SMART trial (6). For example, the average PTV D95% for SMART was 37.1 ± 7.0 Gy for the initial plan and 36.7 ± 7.6 Gy for the adapted plans. In our study, the PTV D95% was 34.0 ± 6.1 Gy initial and 33.8 ± 5.6 Gy adapted. The PTV D90% was 42.2 ± 7.1 Gy for SMART-adapted plans and 36.2 ± 5.7 Gy for adapted plans in our study. Slightly higher target coverage was obtained in the SMART trial, likely due to the use of breath-hold gating rather than compression and the variation in prescription used at our institution over time. However, these differences are within one standard deviation. The adapted plans at our institution achieved similar coverage as the initial reference plan (33.8 Gy vs. 34.0 Gy for PTV D95%), indicating consistent adaptation. In addition, the average PTV D_max_ was 65.0 ± 4.8 Gy for SMART-adapted plans, 52.7 ± 7.5 Gy for all prescriptions in this study, and 61.7 ± 3.5 Gy for the patients in this study with a prescription of 50 Gy (n = 7). This indicates that the plans in our study have lower hot spots than those in the SMART trial, reiterating the need for standardizing planning techniques for this disease site (8).

Detailed assessment of efficacy is somewhat limited by our small sample size and relatively short follow-up period. However, local control, which is the most direct measure of efficacy following SBRT, was encouraging, with no local progression in 80.8% of patients in our study compared to 71% for the no-surgery group in the SMART trial at 730 days (6). The percentage of patients with potentially related grade 3 or higher GI toxicity was 11.5%, which is similar to the rate observed (11.5%) in the SMART trial for borderline resectable and locally advanced pancreatic cancer (6). Another MR-guided pancreatic study observed grade 3–4 late toxicities in five of 41 (12%) patients receiving hypofractionated treatment (7). In terms of our GI toxicities, one of the patients developed cholangitis, and two patients had GI bleeds, with one patient expiring due to the GI hemorrhage. This patient was initially noted to have a GI bleed 98 days after completion of radiation therapy. He underwent an upper endoscopy, which showed an ulcerated mass in the duodenum, with the biopsy showing predominantly necrotic tissue indeterminate for malignancy. There was no evidence of active bleeding. Colonoscopy showed no source of lower GI bleeding, including in the transverse colon, which was within 3 cm of the PTV. Intermittent melena and hematochezia continued, and the patient required two blood transfusions after being found unconscious in pulseless electrical activity and subsequently passing away. This toxicity may have been related to his prior SBRT, although no clear source of GI bleeding was ever identified.

## Conclusion

In this work, we evaluated our workflow for treating pancreatic SBRT without the use of breath-hold motion management. This method increases the availability of treating pancreatic cancer with MR guidance since access to other motion management strategies (gating and breath hold) may not be available to all clinics. The application of abdominal compression allowed for an increased dose of the PTV. The results indicate that the tumor motion is consistent (within ±5 mm) between the CT and MRI simulation images. In addition, the motion of the liver dome in SI was determined to be an overestimate of tumor SI motion most of the time. However, it was found that the liver dome can successfully be used to ensure consistent respiratory motion among all fractions. Using the liver dome as a surrogate with daily cine MRI was found to be a simple yet effective method of pre-treatment quality assurance to ensure the compression belt was applied correctly and that the patient was breathing in a similar manner to the simulation scans. Adding abdominal compression reduced tumor motion, which allows for smaller margins without the need for breath holds, gating, or active tracking. These techniques enabled isotoxic adaptive treatment planning on the Unity and have resulted in similar toxicity rates and local control as observed in the literature.

## Data Availability

The datasets presented in this article are not readily available because of the waiver of consent. Requests to access the datasets should be directed to JS-A (joel-st-aubin@uiowa.edu).
